# River discharge-related nutrient effects on North Sea coastal and offshore phytoplankton communities

**DOI:** 10.1093/plankt/fbac049

**Published:** 2022-09-09

**Authors:** Elisabeth Groß, Julien Di Pane, Maarten Boersma, Cédric L Meunier

**Affiliations:** Shelf Sea System Ecology, Alfred-Wegener-Institut Helmholtz-Zentrum für Polar- und Meeresforschung, Biologische Anstalt Helgoland, Helgoland 27498, Germany; Shelf Sea System Ecology, Alfred-Wegener-Institut Helmholtz-Zentrum für Polar- und Meeresforschung, Biologische Anstalt Helgoland, Helgoland 27498, Germany; Shelf Sea System Ecology, Alfred-Wegener-Institut Helmholtz-Zentrum für Polar- und Meeresforschung, Biologische Anstalt Helgoland, Helgoland 27498, Germany; FB2, University of Bremen, FB2, Bremen 28359, Germany; Shelf Sea System Ecology, Alfred-Wegener-Institut Helmholtz-Zentrum für Polar- und Meeresforschung, Biologische Anstalt Helgoland, Helgoland 27498, Germany; FB2, University of Bremen, FB2, Bremen 28359, Germany

**Keywords:** extreme weather events, salinity, functional traits, community composition, growth rate

## Abstract

As a result of climate change, an increasing number of extreme weather events can be observed. Heavy precipitation events can increase river discharge which causes an abrupt increase of nutrient-rich freshwater into coastal zones. We investigated the potential consequences of nutrient-rich freshwater pulses on phytoplankton communities from three stations in the North Sea. After incubating the phytoplankton cultures with a gradient of nutrient-rich freshwater, we analyzed changes in community diversity, average cell size, growth rate and elemental stoichiometry. Pulses of nutrient-rich freshwater have caused an increase in the growth rate of the phytoplankton communities at two of the three stations and a decrease in cell size within the taxonomic groups of flagellates and diatoms at all stations, indicating a positive selection in favor of smaller taxa. In addition, we observed a decrease in the molar N:P ratio of the phytoplankton communities. Overall, the response of phytoplankton was highly dependent on the initial community structure at each sampling site. Our study demonstrates that the biomass and functional structure of North Sea phytoplankton communities could be altered by an abrupt increase in river discharge, which could have further consequences for higher trophic levels and short-term food web dynamics in the North Sea.

## INTRODUCTION

Phytoplankton form the basis of most marine food webs. Moreover, they respond directly to environmental change due to their short generation times (Collins *et al*., 2014). Since different phytoplankton species have different environmental preferences, changes in environmental parameters usually cause a restructuring of phytoplankton community compositions (Barton *et al*., 2020; Margalef, 1963; Margalef, 1978). This rearrangement of communities can be observed as natural phytoplankton succession following seasonal environmental change, related to changes in temperature, irradiation and dissolved macro-nutrients availability (Caracciolo *et al*., 2021) or on longer time scales caused by anthropogenically induced global change (Hare *et al*., 2007; Spilling *et al*., 2018). The frequency with which phytoplankton is exposed to variation in abiotic parameters strongly depends on their habitat. Although environmental conditions in the open ocean are often rather stable, phytoplankton inhabiting coastal areas experience frequent fluctuations in many parameters (i.e. temperature, dissolved nutrient concentrations, salinity). This is particularly true for areas subjected to high river discharge, such as the southern North Sea, as they receive large inputs of freshwater and nutrients, most of which are derived from human-related activities (Ducrotoy *et al*., 2000).

The North Sea is bordered by several European countries and is connected to the Atlantic Ocean with a wide opening in the North and a smaller opening through the Dover Strait and the English Channel in the Southwest and to the Baltic Sea in the East. As such, the coastal areas, in particular, in the southern North Sea are heavily influenced by river discharge (Kühn *et al*., 2010; Philippart *et al*., 2000). The annual freshwater input from rivers is strongly variable (Prandle *et al*., 1997; Quante *et al*., 2016). Within a climate change context, in addition to warming and elevated *p*CO_2_, greenhouse gas emissions are also expected to increase the frequency and intensity of extreme weather events (Collins *et al*., 2013). Heavy precipitation events can cause abrupt increases in river discharge, which reduce the salinity and increase the dissolved nutrient concentrations of coastal zones, subsequently affecting biological communities potentially up to dozens of kilometers from the coast (Ducrotoy *et al*., 2000; Núñez-Riboni and Akimova, 2017; Voynova *et al*., 2017).

Although an increase in nutrients can boost phytoplankton growth (Pinckney *et al*., 1999), changes in salinity can be detrimental to phytoplankton cells due to osmotic stress (Kirst, 1990). Previous studies that used salinity as an abiotic stressor reported large changes in phytoplankton communities depending on the salinity used in the experiments. Larson and Belovsky (2013), for example, found that species richness of communities and the abundance of particular species, in this case, green algae, were positively correlated with salinity and Lionard *et al*. (2005) observed that both an increase or a decrease in salinity could have a negative impact on phytoplankton growth depending on the origin of the phytoplankton communities. Different phytoplankton groups within the community were either similarly or differently affected by salinity changes, which indicates that the initial community composition may drive the responses observed. Overall, the effect of salinity changes on phytoplankton depends highly on species identity and the tolerance ranges of these species (Flöder *et al*., 2010). As expected, coastal phytoplankton species are more euryhaline than oceanic species (Balzano *et al*., 2011; Brand, 1984), whereby the osmotic acclimation potential of algal species determines their tolerance to different salinities (D'ors *et al*., 2016).

To understand how changes in environmental conditions can affect the functional structure of phytoplankton communities, trait-based approaches are useful. Functional traits are morpho-physio-phenological characteristics of organisms that influence growth, reproduction and survival (Violle *et al*., 2007). Explicitly defined traits, such as cell size, can be used to understand and predict changes in community composition and, ultimately, ecosystem functioning (Litchman and Klausmeier, 2008; Weithoff and Beisner, 2019). Some traits of phytoplankton cells are master traits since they regulate several physiological characteristics. Cell size, elemental stoichiometry and growth rate, for example, are strongly linked (Litchman and Klausmeier, 2008). The elemental stoichiometry of phytoplankton is not only affected by the availability of dissolved nutrients but also varies among taxonomic groups (Geider and La Roche, 2002; Hillebrand *et al*., 2013; Sterner and Elser, 2002). Moreover, the elemental stoichiometry of phytoplankton can be affected by other abiotic parameters than dissolved nutrients, such as temperature (Yvon-Durocher *et al*., 2017) or through metabolomic processes or cell size (Finkel *et al*., 2010). Furthermore, Hillebrand *et al*. (2013) found that the cellular N:P ratios decreased and became less variable with increasing growth rates. In natural environments, also nutrient acquisition strategies play an important role. Among the different strategies which have been proposed (Sommer, 1984), velocity-adapted species, or *r*-strategists, are likely to benefit most from abrupt increases in nutrient availability as these species, which are often small, have high maximum nutrient uptake rates and high maximum growth rates (Litchman *et al*., 2007). Storage-adapted and affinity-adapted species, or *K*-strategists, on the other hand, are better equipped in environments with low nutrient concentrations after a pulsed nutrient supply (Litchman *et al*., 2009; Litchman and Klausmeier, 2008). Phytoplankton communities originating from different geographic locations typically display different dominant strategies. Areas with high nutrient loads, such as coastal areas, are often dominated by smaller cells due to the selection for maximum exponential growth rates. In contrast, the supply of nutrients at different time intervals and scales can lead to the coexistence of smaller and larger cells, with small cells growing fast after the nutrient pulse and large-cell species with higher storage capacity benefiting later in the period (Litchman *et al*., 2009). Overall, trait-based approaches help understand why some species are positively selected in response to environmental changes, and what the consequences for ecosystem functioning may be (Litchman and Klausmeier, 2008).

Several studies investigated the impact of changing nutrient loads on phytoplankton communities, but these focused mostly on the average annual nutrient inputs into oceans and seas (Radach and Pätsch, 2007; Van Beusekom *et al*., 2019). Fewer studies assessed the effects of abrupt increases in nutrient input (Weisse *et al*., 2016) and changes in salinity (D'ors *et al*., 2016). Given the potential influence of abrupt river discharge on phytoplankton communities through sudden changes in salinity as well as dissolved nutrient concentrations, it is crucial to investigate how phytoplankton communities and their functional traits respond to these events. In addition, it is important to understand the role of the original phytoplankton community structure. This is particularly interesting in the case of area-specific environmental conditions since, for example, coastal areas are generally more variable than offshore areas.

In this study, we exposed natural phytoplankton communities sampled in early May from three different stations in the North Sea with increasing distance from shore to pulses of nutrient-rich freshwater of varying intensity. This allowed us to examine the potential impact of an abrupt increase in river discharge on community structure as well as properties such as growth rate, stoichiometric ratios and cell size. Specifically, we tested the following hypotheses:

- Nearshore phytoplankton communities will react to the pulse of nutrient-rich freshwater in a positive way, indicated by higher growth rates, whereas offshore communities will react negatively to pulses of freshwater.
- Pulses of nutrient-rich freshwater will select for small cells with high maximum growth rates.
- Changes in nutrient concentrations and higher growth rates will lead to a shift in the elemental stoichiometry of phytoplankton communities and result in decreasing cellular N:P ratios.

## MATERIALS AND METHODS

### Experimental setup

To assess the potential impact of an abrupt increase in river discharge on phytoplankton communities, we conducted an experiment testing three levels of decreased salinity and increased dissolved nutrient concentrations on natural phytoplankton communities from three stations in the southern North Sea. As response variables, we focused on the growth rate of the community, shifts in community composition and size structure, as well as elemental stoichiometry.

The sampling and experiment were conducted on board the research vessel Pelagia of the Royal Netherlands Institute for Sea Research (NIOZ). Seawater containing natural phytoplankton communities was collected from three stations (st1: 53° 11.23′ N, 4° 47.67′ E; st2: 53° 39.88′ N, 4° 03.07′ E; st3: 54° 49.02′ N, 3° 45.98′ E) along a gradient from the Dutch coast to the open North Sea from 8th to10th May 2019 (Supplementary Fig. S1). The water was sampled from 2 m depth by a rosette sampler equipped with CTD sensing (Sea-Bird SBE 9, Sea-Bird Electronics, Bellevue, Washington) and 24 12-L Niskin bottles. Twenty liters of water were sieved through a 200 μm mesh to exclude larger grazers and were subsequently sampled for initial dissolved nutrient concentrations, particulate organic carbon (POC), nitrogen (PON) and phosphorus (POP), as well as phytoplankton community composition. The remaining water was used to conduct the experiments. Smaller grazers or parasites could not be excluded from the communities. To test the influence of different intensities of pulses of nutrient-rich freshwater, simulating an abrupt increase in river discharge, we used a freshwater medium that was added to the phytoplankton communities before incubation. The medium was prepared according to f/2 medium (Guillard and Ryther, 1962), including silicate (SiO4-), using MilliQ water. Cell culture flasks (Falcon) of 910 mL were filled in triplicates to 10, 20 and 30% with freshwater medium and topped up with sieved seawater containing natural phytoplankton communities for the treatments with 10, 20 and 30% addition of nutrient-rich freshwater (trt10, trt20, trt30), respectively. Salinity decreased by about three to eight units from the lowest to the most severe treatment (Table I). Similar salinity changes after flood events with high river discharges were observed in the southern North Sea (Voynova *et al*., 2017). The fourth group of bottles was filled with sieved seawater only and served as a control group. This experiment resulted in 3 (regions) × 4 (treatments) × 3 replicates, for a total of 36 experimental flasks. Nutrient concentrations for each treatment can be found in Table I. Increasing treatment intensity caused an increase in osmotic stress due to greater salinity changes but at the same time delivered larger amounts of dissolved inorganic nutrients for phytoplankton growth. The flasks were incubated on board for 72 h with simulated natural light under a 16:8 day:night cycle, which is approximately the day:night duration on Texel, Netherlands, at the beginning of May, and irradiance of 90 μmol photons m^−2^ s^−1^ (GHL Mitras Lightbar 2 Daylight, 6 500 K, dimmed to 80%) in a temperature-constant container at 10°C, simulating the mean water temperature at the three stations. All cell culture flasks were carefully homogenized manually several times a day to prevent sedimentation of phytoplankton cells.

**Table I:** Dissolved inorganic nitrogen *(DIN), phosphorus (DIP) and silicate (DSi) in μmol L^−1^ before and after 72-h incubation with nutrient-rich freshwater in four treatments at three stations*

		Initial			Final			Initial		Final		
		DIN μmol L^−1^	DIP μmol L^−1^	DSi μmol L^−1^	DIN μmol L^−1^	DIP μmol L^−1^	DSi μmol L^−1^	TN μmol L^−1^	TP μmol L^−1^	TN μmol L^−1^	TP μmol L^−1^	Salinity
Station 1												
	ctrl	3.6	0.9	4.4	4.8	0.7	4.2	19.9	1.3	11.3	1.0	31.5
	trt10	77.8	2.4	6.4	75.3	2.1	6.3			87.8	2.8	28.7
	trt20	104.0^*^	3.9	8.6	99.3^*^	3.3	7.8			111.4^*^	3.9	26.2
	trt30	111.1^*^	4.7	9.5	110.7^*^	5.0	10.6			119.4^*^	5.6	24.3
Station 2												
	ctrl	3.2	0.9	1.8	3.2	0.7	1.8	5.9	1.1	7.7	0.8	31.6
	trt10	72.9	2.0	4.3	75.6	2.1	4.8			81.8	2.5	28.3
	trt20	104.9^*^	3.9	7.6	102.3^*^	3.6	6.8			107.7^*^	4.0	26.4
	trt30	115.1^*^	5.6	10.3	109.0^*^	4.7	8.4			113.3^*^	5.1	24.2
Station 3												
	ctrl	3.4	0.8	0.6	2.9	0.6	0.5	5.9	0.9	7.3	0.8	34.5
	trt10	78.3	2.3	4.3	76.4	2.2	4.2			86.3	2.8	31.4
	trt20	99.3^*^	3.3	5.8	98.5^*^	3.4	5.8			108.2^*^	4.0	28.6
	trt30	110.9^*^	5.1	8.9	110.4^*^	5.0	9.0			116.7^*^	5.6	26.3

Total nitrogen (TN, DIN + PON) and total phosphorus (TP, DIP + POP) in μmol L^−1^ at each station before and after 72 h of experimental incubation in four treatments. Numbers marked with a “^*^” describe values above the calibration curve. Data are means of three replicates.

### Growth rate

To calculate the growth rate of phytoplankton communities, we used POC, representing the biomass in μg L^−1^. Final POC samples were taken from each cell culture flask. To avoid using a large volume of each flask at the start of the experiment, we used one set of replicates with only sieved seawater as the initial sample. Considering that the sampled communities were diluted by 10, 20 and 30% depending on the treatment before the experiment, the initial POC content was multiplied by 0.9, 0.8 and 0.7, respectively, before calculating the growth rate μ (day^−1^) using the following equation:

$$ \mu =\ln \left({N}_t/{N}_0\right)\bullet \Delta{t}^{-1} $$

whereby *N*_0_ and *N_t_* describe the initial and final POC (μg C L^−1^) and Δ*t* (Day) is 3 in this case since the experiment ended after 72 h.

In addition, we calculated the absolute change in growth rate relative to the control from each treatment.

### Community analysis

Initial and final samples for phytoplankton community composition were obtained by fixing 200 mL of water from the incubation flasks with acidic Lugol’s iodine solution (final concentration 0.5%). Phytoplankton cells were counted using the Utermöhl method (Utermöhl, 1958) and identified to genus or species level when possible, otherwise grouped into size-dependent groups or “morphotypes”. To assess changes in community composition when exposed to our treatments, we used the most abundant taxa having a cumulative abundance of up to 95%. This was done for clarity and to avoid focusing on a large number of taxa that make up only a small percentage of the frequently occurring taxa. The biovolume of each taxonomic unit was calculated using cell dimensions and geometric formulas (Hillebrand *et al*., 1999) and used to assess changes in the size structure of the phytoplankton communities. We used the entire community to calculate the community-weighted mean (CMW) cell volume.

### Particulate and dissolved nutrient analyses

To assess the impact of the treatments on the elemental stoichiometry of phytoplankton communities, samples of 200 mL were filtered at the onset and the end of the incubation period on pre-combusted and pre-washed GF/F glass microfiber filters (Whatman, 25 mm, pore size 0.7 μm). All filters were stored individually in Eppendorf tubes at −20°C. For POC and PON analyses, filters were dried for at least 48 h at 60°C in a drying chamber before being wrapped in tin foil and analyzed with an elemental analyzer (Elementar vario MICRO cube). Particulate P was measured by spectrometric determination (Thermo Scientific Multiskan Spectrum) of orthophosphate (Grasshoff *et al*., 1999). Dissolved inorganic nitrogen (DIN), phosphorus (DIP) and silicate (DSi) were measured at the beginning of the experiment and after the 72-h incubation period to evaluate nutrient changes in our treatments. A volume of 50 mL was filtered through nylon membrane filters (Merck, 47 mm, pore size 0.2 μm) and stored at −20°C. The samples were measured with an autoanalyzer (Alliance Instruments GmbH) after a modified method after Grasshoff (Seal/Alliance methods; (Grasshoff *et al*., 1999).

### Data analysis

The software R (R core team, 2021) was used for all statistical analyses and to draw the figures. For the community analyses, we used the most abundant taxa with a cumulative abundance of 95%. This threshold was chosen to compromise the representativeness and clarity of the graphic representation. To calculate the Community-Weighted Mean (CWM) cell size, we used the packages *FD, tidyr* in RStudio. Package *plyr* was used to calculate the mean and standard deviation of the data set. Package *ggplot2* was used to draw the graphs. Differences in CMW cell volume, growth rate and elemental stoichiometry between the treatments and stations were tested using two-way analyses of variance (ANOVA) and Tukey’s post hoc comparison. R packages for statistical analysis were *carData* and *car*. For all statistical comparisons, normality (Shapiro) and variance homogeneity (Levene) tests were performed. Data were transformed (square root) if criteria of a normal distribution or variance homogeneity were not met. The threshold of significance was set at 5% for all analyses.

## RESULTS

### Growth rate

**Fig. 1:**
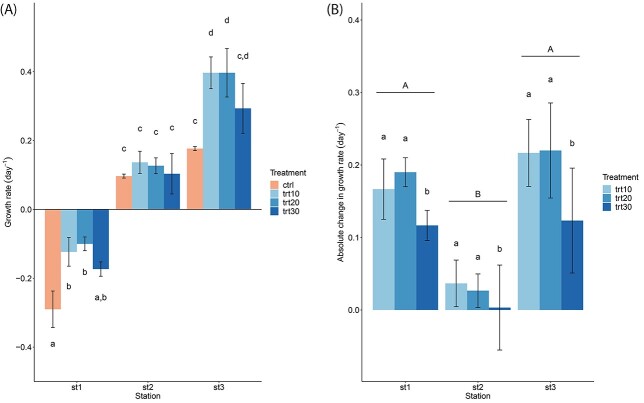
Influence of nutrient-rich freshwater pulses (10, 20 and 30%) on the growth rate (**A**) and the absolute change in growth rate relative to the control (**B**) of phytoplankton communities at three stations over a 72-h incubation. Data presented are means and standard deviations of three replicates. Different letters above bars indicate significant differences (2-way ANOVA, Tukey post hoc test, *P* < 0.05). Capital letters (A, B) indicate significant differences between stations, and small letters (a, b) indicate a significant difference between treatments within one station.

The growth rates at Station 1 varied between −0.29 ± 0.053 and − 0.10 ± 0.020 day^−1^, Station 2 values ranged from 0.10 ± 0.059 to 0.14 ± 0.032 day^−1^ and Station 3 from 0.18 ± 0.006 to 0.40 ± 0.070 day^−1^ (Fig. 1**A**). A two-way ANOVA revealed significant differences between stations and treatments as well as a statistically significant interaction between the effects of station and treatment (Table II). Overall, the treatments had a positive effect on the growth rate of phytoplankton communities compared with the control (Tukey’s post hoc test, p_trt10_ ≤ 0.001, p_trt20_ ≤ 0.001, p_trt30_ = 0.0036). In addition, growth rates in treatments trt10 and trt20 were significantly higher than those in trt30 (Tukey’s post hoc test, p_trt10_ = 0.027, p_trt20_ = 0.017). Within Station 2, we did not observe significant differences between the treatments (Tukey’s post hoc test, *P* ≥ 0.99). Within Station 1 and Station 3, however, the pulse of nutrient-rich freshwater in treatments trt10 and trt20 led to a significant increase in growth rates (Tukey’s post hoc test, *P* ≤ 0.0041). No significant differences were found between the most severe treatment trt30 and the control at Station 1 and Station 3 (Tukey’s post hoc test, *P* ≥ 0.098). Although the growth rates at Station 1 and Station 3 were significantly different, the absolute change in growth rate due to the treatments was similar (Fig. 1**B**). A two-way ANOVA revealed significant differences between stations (2-way ANOVA, *F*_2,18_ = 32.46, *P* < 0.001) and treatments (2-way ANOVA, *F*_2,18_ = 5.37, *P* = 0.015). Overall, the absolute change in growth rate due to the treatments was at Station 1 and Station 3 significantly higher than at Station 2 (Tukey’s post hoc test, p_st1_ < 0.001, p_st3_ < 0.001). Within Station 1 and Station 3, trt10 and trt20 caused significantly higher growth rates than trt30 (Tukey’s post hoc test, p_trt10_ = 0.037, p_trt20_ = 0.022).

**Table II:** Two-way analysis of variance, with growth rate (day^−1^) of phytoplankton communities as the dependent variable and station and treatment as independent variables

Effect	df_1_, df_2_	*F*	*P*
Growth rate			
Station	2, 24	380.39	<0.001^***^
Treatment	3, 24	22.36	<0.001^***^
Station × treatment	6, 24	3.47	0.013^*^

Significant differences (*P* < 0.05) are highlighted in bold

### Taxonomic structure and cell size

**Fig. 2:**
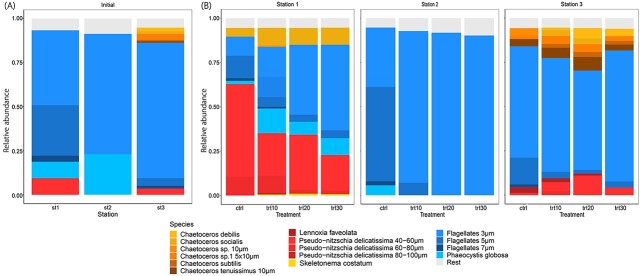
Relative abundances of the most abundant taxa at the three stations before incubation with nutrient-rich freshwater (**A**) and the influence of nutrient-rich freshwater pulses (10, 20 and 30%) on the phytoplankton community composition at three stations after a 72-h incubation (**B**). Taxa shown are the most abundant contributing to 95% of the communities.

We analyzed the relative abundance of the phytoplankton taxa between stations. The initial communities sampled at three stations in the North Sea in early May were dominated by flagellates, which we grouped into cell sizes of 3 μm, 5 μm and 7 μm (Fig. 2**A**). At Station 1, closest to the coast, flagellates between 3 and 7 μm contributed 74.6% of the relative abundance of cells in the community, with 42.5% being flagellates of 3 μm cell size. In addition, colonies of *Phaeocystis globosa* and cells of the diatom species *Pseudo-nitzschia delicatissima* contributed 9% each to the phytoplankton community at Station 1. At Station 2, only flagellates of 3 μm cell size and *P. globosa* contributed to 95% of the relative abundance of the community with 68.3 and 23.0%, respectively. The initial community at Station 3 was also dominated by flagellates with cell sizes between 3 and 7 μm, which contributed to 82.8% of the relative abundance, of which 76.9% were cells of size 3 μm only. Several *Chaetoceros* species built up 8.6% of the relative abundance of the community, and *P. delicatissima* contributed with 4.3%.

After the 72-h incubation, a shift in community structure was observed at all stations, with the most pronounced changes occurring at Station 1 and Station 3 (Fig. 2**B**). Comparing the control from Station 1 after 3 days of incubation with the initial sample, the diatom species *P. delicatissima* was much more abundant after the incubation with the addition of nutrient-rich freshwater, forming 62.8% of the relative abundance of cells in the phytoplankton community with 52.2% smaller cells of 40–60 μm and 10.6% cells of 60–80 μm cell size. Also, cells of the diatom species *Chaetoceros socialis* contributed to 4.9% of the community. The strong shift in community composition was caused by a simultaneous decrease in flagellates and an increase in *P. delicatissima* (Supplementary Fig. S2**A**). However, as treatment intensity increased, the relative abundance of diatoms decreased from 67.7% in the control group to 32.4% in the most severe treatment trt30 due to a large increase in flagellates, including *P. globosa*. Within the group of flagellates as well as for *P. delicatissima*, we observed a shift towards smaller size groups. At Station 2, we observed a similar pattern in terms of cell size change in response to treatment intensity. Although 33.6% of the relative abundance of cells in the community was formed by 3-μm flagellates and 53.1% by 5-μm flagellates in the control, flagellates of 3 μm size increased with treatment intensity (Supplementary Fig. S2**B**) and built 95% of the cumulative abundance of the phytoplankton community in treatments trt20 and trt30. At Station 3, the pulse of nutrient-rich freshwater led to an increase in the relative abundance of diatom species from 14.8 in the control to 36.3% in trt20. The group of diatoms included *P. delicatissima* of different cell sizes as well as several *Chaetoceros* species. Although the relative abundance of flagellates decreased with treatment intensity, it was almost exclusively flagellates of larger size, and the relative abundance of 3-μm flagellates was not strongly affected (Supplementary Fig. S2**C**). The community structure in the most severe treatment was similar to the control, except with a greater relative abundance of flagellates with a 3 μm cell size.

**Fig. 3:**
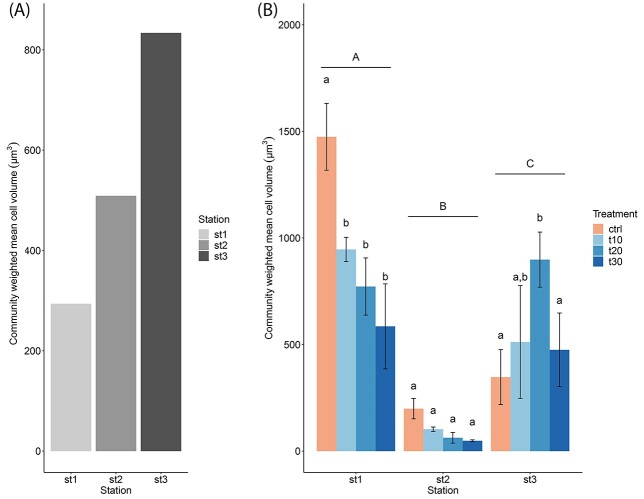
CWM cell volume (μm^3^) of phytoplankton communities at the three stations before incubation with nutrient-rich freshwater (**A**) and the influence of nutrient-rich freshwater pulses (10, 20 and 30%) on the CWM cell volume (μm^3^) of phytoplankton communities at three stations after a 72-h incubation (**B**). Data presented are means and standard deviations of three replicates. Different letters above bars indicate significant differences (2-way ANOVA, Tukey post hoc test, *P* < 0.05). Capital letters (A, B, **C**) indicate significant differences between stations, and small letters (a, b) indicate a significant difference between treatments within one station. Significant differences in interactions between station and treatment can be found in Supplementary Table S1.

In addition to the taxonomic structure, we assessed the CMW cell volume to investigate potential shifts. Contrary to what the relative abundance would suggest, in the initial samples, we observed the lowest average cell volume at Station 1, closest to the coast, and the highest at Station 3, the most offshore station (Fig. 3**A**). However, the CWM cell volume at Station 1 and Station 2 changed drastically after the 72-h incubation with nutrient-rich freshwater. At Station 1, the CMW cell volume increased from initially about 290 μm^3^ to about 1470 μm^3^ in the control and 590 μm^3^ in trt30 (Fig. 3**B**), suggesting a positive selection of larger cells. At Station 2, the CWM cell volume decreased from initially about 510 μm^3^ to about 200 μm^3^ in the control and 50 μm^3^ in trt30. At Station 3, we observed an overall decrease in CWM cell volume from initially about 830 μm^3^ to about 350 μm^3^ in the control. The incubation with nutrient-rich freshwater in the least severe and the intermediate treatments caused an increase in CWM cell volume to about 510 μm^3^ in trt10 and 900 μm^3^ in trt20. These changes in the CWM cell volume align with the changes in relative abundance we observed for the phytoplankton communities due to the different treatments.

### Elemental stoichiometry

**Fig. 4:**
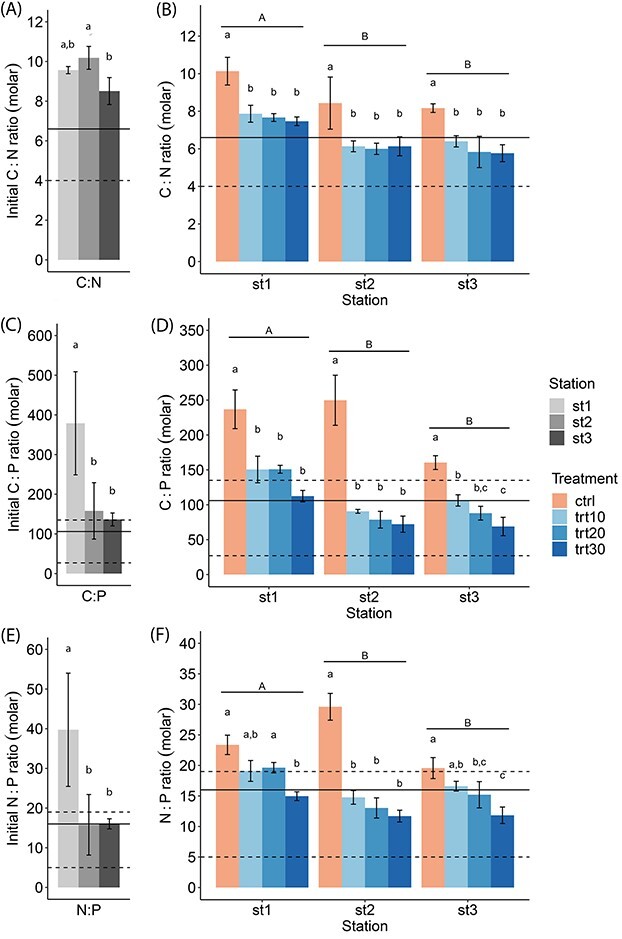
Molar C:N (**A**), C:P (**C**) and N:P (**E**) ratios of initial community samples from three stations and the influence of nutrient-rich freshwater pulses (10, 20 and 30%) on the molar C:N (**B**), C:P (**D**) and N:P (**F**) ratios of phytoplankton communities at three stations after a 72-h incubation. Data presented are means and standard deviations of three replicates. Horizontal lines represent the Redfield ratio of C:N:P = 106:16:1. The area between dashed lines represents C:N, C:P and N:P ratios of phytoplankton growing under optimal nutrient-replete conditions (Geider and La Roche, 2002). In (A), the upper limit is C:N = 17. Different letters above bars indicate significant differences (2-way ANOVA, *P* < 0.05). Capital letters (A, B) indicate significant differences between stations, and small letters (a, b) indicate significant differences between treatments within one station. Significant differences in interactions between station and treatment can be found in Supplementary Table S2.

We measured POC, PON and POP (Supplementary Fig. S3) and calculated the molar ratios of C:N, C:P and N:P for initial samples (Fig. 4**A**,**C** and **E**) and after 72-h incubations (Fig. 4**B**,**D** and **F**). In addition, we used the Redfield ratio (C:N:P = 106:16:1) and ranges of molar ratios reported by Geider and La Roche (2002) to assess possible nutrient limitations. Under optimal nutrient-replete conditions, values of cellular N:P range from 5 to 19, values of cellular C:N range from 3 to 17 and values of cellular C:P range from 27 to 135. The initial C:P ratios that were measured at Station 1 ranged from 292 to 528, and the initial N:P ratios ranged from 31 to 59, which both indicate a strong initial P-limitation at the coastal station. A one-way ANOVA revealed significant differences between stations for the initial C:N ratio (1-way ANOVA, *F*_2,6_ = 7.78, *P* = 0.022), C:P ratio (1-way ANOVA, *F*_2,6_ = 6.46, *P* = 0.032) and N:P ratio (1-way ANOVA, *F*_2,6_ = 7.78, *P* = 0.022). Overall, the initial C:P and N:P ratios were significantly higher at Station 1 than at Station 2 and Station 3 (Tukey’s post hoc test, p_C:P_ ≤ 0.046, p_N:P_ ≤ 0.048).

Overall, the pulses of nutrient-rich freshwater led to a decrease in cellular C:N, C:P and N:P ratios of phytoplankton communities at all stations, and we identified significant differences between the stations and treatments as well as in their interaction (2-way ANOVA, Table III). The molar C:N ratio of the phytoplankton communities ranged between 10.1 ± 0.7 in the control at Station 1 and 5.8 ± 0.5 in trt30 at Station 3 and therefore did not indicate a N-limitation. At all stations, the molar C:N ratio in the treatments with nutrient-rich freshwater was significantly lower than in the controls (Tukey’s post hoc test, *P* ≤ 0.05). However, we did not observe a significant difference between the treatments trt10, trt20 and trt30 (Tukey’s post hoc test, *P* ≥ 0.124). For both the molar C:P ratio and the molar N:P ratio of the communities, the controls exceeded the ratio of 135 mol C:mol P and 19 mol N:mol P at all stations, indicating P-limitation. The molar C:P ratio in the controls ranged between 249.7 ± 35.9 at Station 2 and 160.4 ± 9.9 at Station 3. The molar N:P ratio in the controls ranged between 29.6 ± 2.2 at Station 2 and 19.6 ± 1.7 at Station 3. From control to treatment trt10, a significant decrease in molar C:P was observed at all stations (Tukey’s post hoc test, *P* ≤ 0.046). At Station 1 and Station 2, no significant differences were observed between treatments trt10, trt20 and trt30 (Tukey’s post hoc test, *P* ≥ 0.163). Only at Station 3, the molar C:P ratio in trt30 was significantly lower than in trt10 (Tukey’s post hoc test, *P* = 0.006). For the molar N:P ratio, only at Station 2, the control was significantly higher than the molar N:P ratio in trt10 (Tukey’s post hoc test, *P* ≤ 0.001). At all stations, the molar N:P ratio in the most severe treatment trt30 was significantly lower than the controls (Tukey’s post hoc test, *P* ≤ 0.001).

**Table III:** Two-way analysis of variance, with the molar ratios (C:N, C:P, N:P) of phytoplankton communities as the dependent variable and station and treatment as independent variables

Effect	df_1_, df_2_	*F*	*P*
C:N ratio			
Station	2, 24	31.96	<0.001^***^
Treatment	3, 24	35.10	<0.001^***^
Station × treatment	6, 24	0.22	0.97
C:P ratio			
Station	2, 24	49.05	<0.001^***^
Treatment	3, 24	103.99	<0.001^***^
Station × treatment	6, 24	6.16	<0.001^***^
N:P ratio			
Station	2, 24	17.55	<0.001^***^
Treatment	3, 24	55.34	<0.001^***^

Significant differences (*P* < 0.05) are highlighted in bold

## DISCUSSION

We hypothesized that nearshore phytoplankton communities would respond positively to the pulses of nutrient-rich freshwater, whereas offshore communities respond negatively to pulses of nutrient-rich freshwater, which our results did not fully confirm. Compared with the initial biomass, we observed a negative growth rate for the phytoplankton community closest to the coast and a positive growth rate for the most offshore station. Representative of the high productivity of coastal waters (Cloern *et al*., 2014), we also observed about six times higher initial biomass at Station 1, which is closest to the coast, compared with the other two stations (Supplementary Fig. S4), as well as the highest amount of total nitrogen and phosphorus (Table I). Thus, the high initial biomass could be a possible explanation for the loss of biomass at Station 1, which may not have been able to be sustained in our experimental setup. However, the addition of nutrient-rich freshwater at Station 1 had a positive effect on the growth rate in comparison to the control and attenuated the negative effect of the incubation, similar to Station 3. At both stations, the least severe and intermediate treatment caused a significant increase in biomass compared with the control. For the most severe treatment, no significant increase in growth rates could be observed. These results suggest that the positive effect the nutrients have when phytoplankton communities are subjected to pulses of nutrient-rich freshwater might only be the case until salinity changes cause osmotic stress in phytoplankton cells. In contrast, the second station showed no change in growth rate regardless of treatment. At the same time, the stations also differed in their community composition. Although for Station 1 and Station 3, both flagellates and diatoms contributed to 95% of the relative abundance, at Station 2, only flagellates represented 95% of the phytoplankton community. This strongly indicates that initial diversity plays an important role for phytoplankton communities when facing abrupt changes in environmental conditions, supporting the diversity-stability hypothesis (Corcoran and Boeing, 2012; Macarthur, 1955; Otero *et al*., 2020).

In terms of the initial community structure at Station 1, flagellates comprised the most abundant taxa. The relatively high nutrient loads in coastal areas should generally select smaller, velocity-adapted species (Litchman *et al*., 2009), as we could also observe for the initial sample at Station 1. Under the absence of fresh nutrients, larger, affinity-adapted species with storage capacities generally succeed (Litchman and Klausmeier, 2008), which we could observe in the control group of Station 1, where the absolute and relative abundance of flagellates decreased within the 72-h incubation and the abundance of diatoms, especially the species *P. delicatissima*, increased. In addition, initial silicate and nitrogen concentrations were relatively high compared with Station 2 or Station 3, which could have benefitted the growth of diatoms. This increase in diatoms was also likely the cause for the increase in CWM cell volume of the phytoplankton communities after incubation. In contrast, exposure to pulses of nutrient-rich freshwater caused an increase in the abundance of flagellates at Station 1, and within the flagellate group, a shift towards smaller size classes. These results support the hypothesis that small, fast-growing taxa generally benefit most from abrupt increases in dissolved nutrient concentrations as they have high maximum nutrient uptake rates and high maximum growth rates (Litchman and Klausmeier, 2008; Sommer, 1984).

At Station 2, the initial phytoplankton community was dominated by unicellular flagellates between 3 and 7 μm, as well as by the species *P. globosa*. Although no significant changes in biomass were observed between treatments at Station 2, located about 70 km away from Station 1, we also observed a strong increase in small flagellates in response to pulses of nutrient-rich freshwater for this station. In addition, the decline in CWM cell volume after incubation was likely due to the loss of the diatom species *Rhizosolenia imbricata*, which occurred at a very low absolute abundance of about 0.6% in the initial sample, but had a substantial influence on CWM cell volume due to its large size. We hypothesize that the overall lower biomass and diversity of phytoplankton communities at this offshore station compared with the coastal Station 1 are related to the greater distance from the coast and hence lower nutrient availability (Table I) (Burson *et al*., 2016; Stelfox-Widdicombe *et al*., 2004).

At Station 3, located close to the Dogger Bank and with the greatest distance from the coast, the pulses of nutrient-rich freshwater increased diatom abundances, both those of small diatoms of the genus *Chaetoceros* as well as larger diatoms of the species *P. delicatissima*. This increase in diatoms, combined with the low dissolved Si concentrations we measured at Station 3, as well as the increase in phytoplankton growth rate after the pulses of nutrient-rich freshwater, indicate that phytoplankton growth was likely Si-limited. At the same time, the Dogger Bank region is known for high year-round primary production (Kröncke and Knust, 1995). Nutrient upwelling in this area and associated fluctuations in nutrient availability may have been the cause of the coexistence of taxa with smaller and larger cell sizes and their respective strategies for nutrient acquisition, as we observed for the initial community structure at Station 3. Since only intermediate intensities of nutrient-rich freshwater pulses promoted phytoplankton growth, especially that of flagellates and diatoms, we hypothesize that these organisms benefitted from the addition of nutrients but were not negatively affected by an enhanced drop in salinity in the least severe and intermediate treatment.

Our results suggest that the effects of an abrupt increase in nutrient-rich freshwater due to heavy precipitation events and an increased river discharge on marine phytoplankton communities depend largely on the initial community structure. Although increases in dissolved nutrients significantly enhanced primary production at two of three stations, phytoplankton taxa did not seem to suffer from changes in salinity, as growth rates in the treatments were higher or similar to those in the control. Also, Shikata *et al*. (2008), who investigated the effect of rapid changes in salinity on the growth of diatoms and flagellates isolated from the field, did not observe changes in growth between salinities of 15 and 32. Although we found no difference between the control and the most severe treatment in terms of growth rate, suggesting no strong negative effects, phytoplankton did not benefit from nutrient additions when a more drastic decrease in salinity accompanies these. In addition, we observed at all stations a decrease in cell size for flagellates as well as for diatoms, which supports our second hypothesis that pulses of nutrient-rich freshwater favor smaller, fast-growing species (Sommer, 1984). However, working with natural plankton communities, we could not separate autotrophic and heterotrophic species of the same cell size but excluded only cells larger than 200 μm. For this reason, we cannot exclude the possibility that microzooplankton grazing played a role during the incubation period and affected the phytoplankton community composition.

Former studies on the impact of increased river discharge mainly focused on the input of nutrients and terrestrial matter, which can potentially lead to light limitation. Deininger *et al*. (2016) observed an increase in biomass after adding soil and connecting nutrients to natural phytoplankton communities in a mesocosm experiment, with diatom abundance increasing immediately after the addition of soil followed by an increase in dinoflagellates a few days later. Although in the study of Deininger *et al*. (2016), no negative effect of the browning of water was observed, Frigstad *et al*. (2020) stressed the importance of light limitation as high river discharge is also connected to an input of terrestrial matter. Our study suggests that especially small flagellates of 3 μm cell size benefitted from the addition of nutrient-rich freshwater. Taking potential light limitation into account, small cells could not only have an advantage over larger cells when subjected to pulses of nutrient-rich freshwater but also under light limitation since they are less affected by low light availability in comparison to larger cells (Litchman *et al*., 2010). At the same time, however, an overall decrease in phytoplankton cell size could promote the growth of heterotrophic dinoflagellates or ciliates as microzooplankton is more efficient in consuming small prey than mesozooplankton, which lengthen the food chain and lead to carbon loss (Aberle *et al*., 2015; Fenchel, 2008). As mentioned previously, our study design did not allow the separation between autotrophic, mixotrophic or heterotrophic flagellates. Since a great number of flagellates are mixotrophs, a shift in phytoplankton communities towards flagellates might also impact the energy transfer to higher trophic levels. As Ward and Follows (2016) found, mixotrophy can enhance biomass transfer to larger organisms at higher trophic levels because energy and biomass can enter the food web across multiple trophic levels.

Although phytoplankton growing at steady-state conditions in the laboratory can achieve a constant cellular C:N:P ratio, this is nearly impossible in the ocean due to short- and long-term changes in abiotic conditions (Moore *et al*., 2013). In our study, we hypothesized that the elemental stoichiometry of the phytoplankton community near the coast would shift and that the N:P ratio would decrease because pulses of nutrient-rich freshwater would promote phytoplankton growth rates, which leads to a more restricted and overall lower cellular N:P ratio (Hillebrand *et al*., 2013). The particulate N:P ratio of the phytoplankton community at Station 1, closest to the coast, indicated a strong P limitation, which was also reported by Burson *et al*. (2016), and no strong nutrient limitation at Stations 2 and 3. The particulate molar N:P ratio has widely been used to estimate whether phytoplankton growth is likely N- or P-limited (Lenton and Watson, 2000; Tyrrell, 1999). Although Geider and La Roche (2002) reviewed that the values of cellular N:P ratios for phytoplankton growing under nutrient-replete conditions have a certain degree of variation, with N:P values under nutrient-replete conditions being below the Redfield ratio of 16, Hillebrand *et al*. (2013) indicated that variation in N:P decreases with increasing growth rate, and converges towards 16. In support of our hypothesis that pulses of nutrient-rich freshwater may alleviate nutrient limitation, we observed that our treatments resulted in decreased cellular C:N, C:P and N:P ratios, which converged towards values in a range that was reported for non-limited growth conditions (Geider and La Roche, 2002). Similar to cell size, elemental stoichiometry of phytoplankton can also be considered a master trait, as it links environmental conditions to growth rates and food web interactions (Finkel *et al*., 2010). The C:N:P signature of phytoplankton cells is an important determinant of their nutritional quality for higher trophic levels. A reduction in nutrient quality due to changes in the elemental stoichiometry of phytoplankton, for example, could alter secondary production (Boersma and Elser, 2006; Malzahn and Boersma, 2012) while grazing pressure increases as predators need to change their feeding behavior to meet their nutrient requirements (Hessen *et al*., 2013; Vad *et al*., 2020). Consequently, by substantially and rapidly modifying the elemental stoichiometry of phytoplankton communities, abrupt nutrient-rich freshwater inputs may have important short-term effects on food web processes.

## CONCLUSION

In this study, we investigated the possible effects of abrupt river discharge by subjecting natural phytoplankton communities from the North Sea to pulses of nutrient-rich freshwater. We found that initial community composition plays an important role in response to abrupt changes in dissolved nutrient concentration and salinity. The more diverse communities near the coast and the Dogger Bank responded with an increase in biomass following pulses of nutrient-rich freshwater, whereas the community sampled between these stations was less affected in terms of community growth rate. At the same time, we did not observe any strongly negative impact of decreasing salinity, indicating a large salinity tolerance of North Sea phytoplankton communities, even though it appears that although phytoplankton benefit from nutrient additions, this is no longer the case when accompanied by a more drastic decrease in salinity. Furthermore, we demonstrate that pulses of nutrient-rich freshwater can lead to an overall decrease in cell size and an increasing abundance of small-celled flagellates. Together with the observed shifts in the elemental stoichiometry of phytoplankton, changes in community structure and cell size associated with extreme weather events could modulate short-term responses in higher trophic levels as they are exposed to sudden changes in their food sources.

## Supplementary Material

Supplementary_material_fbac049
