# Supplementary material for: River discharge-related nutrient effects on North Sea coastal and offshore phytoplankton communities
Source: J Plankton Res. 2022 Sep 9;44(6):947–60. doi: 10.1093/plankt/fbac049 (PMC9692191; doi:10.1093/plankt/fbac049)
Supplement: Supplementary_material_fbac049 [file supplementary_material_fbac049.docx]

**Supplementary material**

Figure *S*1: Location of the transect with the three sampling stations in the North Sea. Samples were taken from 8^th^ to 10^th^ May at the stations with the coordinates: st1: 53° 11.23' N, 4° 47.67' E; st2: 53° 39.88' N, 4° 03.07' E; st3: 54° 27.42' N, 3° 13.90' E

Figure *S*2: Absolute abundances of the most abundant taxa at station 1 (A), station 2 (B), and station 3 (C) before and after a 72-hour incubation with nutrient-rich freshwater pulses (10, 20, and 30%). Taxa shown are the most abundant contributing to 95 % of the community.

Figure *S*3: Particulate organic carbon (A), nitrogen (B), and phosphorus (C) of initial community samples from three stations. Data presented are means and standard deviations of three replicates. Different letters (a, b) indicate significant differences (two-way ANOVA, p < 0.05).

Table *S*1: Results of Tukey’s *post-hoc* comparison after two-way ANOVA of average cell volume of phytoplankton communities after 72-hour incubation with interactions between stations (st1, st2, st3) and treatments (ctrl, trt10, trt20, trt30) with an initial dilution with nutrient-rich freshwater by 10, 20, and 30 %. The symbol ‘+’ indicates a significant difference (Tukey’s *post-hoc* comparison, p < 0.05), and the symbol ‘-’ indicates the absence of a significant difference.

Table *S*2: Results of Tukey’s *post-hoc* comparison after two-way ANOVA of molar C:N, C:P, and N:P ratios of phytoplankton communities after 72-hour incubation with interactions between stations (st1, st2, st3) and treatments (ctrl, trt10, trt20, trt30) with an initial dilution with nutrient-rich freshwater by 10, 20, and 30 %. The symbol ‘+’ indicates a significant difference (Tukey’s *post-hoc* comparison, p < 0.05) and the symbol ‘-’ indicates the absence of a significant difference.
